# Drug and pesticide usage for sea lice treatment in salmon aquaculture sites in a Canadian province from 2016 to 2019

**DOI:** 10.1038/s41598-022-08538-w

**Published:** 2022-03-16

**Authors:** Dounia Hamoutene, Vanessa Oldford, Sebastien Donnet

**Affiliations:** 1 Saint Andrews Biological Station, Fisheries and Oceans Canada, 125 Marine Science Drive, St. Andrews, NB E5B 0E4 Canada; 2 Northwest Atlantic Fisheries Center, Fisheries and Oceans Canada, P.O. Box 5667, St. John’s, NL A1C 5X1 Canada

**Keywords:** Environmental sciences, Ocean sciences

## Abstract

We used 4 years of publicly available data (2016–2019) on chemical usage at salmon sites with information on production, stocking, locations and environmental conditions to explore patterns of anti-sea lice treatments in a Canadian province. Results show that sequential chemical treatments are prevalent, emamectin benzoate (EMB) with azamethiphos being the most used combination with a decrease in ivermectin usage. Relatively high rates of usage of EMB per fish biomass may point to potential lice resistance patterns with information needed on mechanisms and local populations. Lower or no chemical usage at some sites indicate less sea lice infestations likely influenced by localized site conditions (coves), and a lessened need for medication due to the usage of cleaner fish and possibly other non-chemical methods (not documented in this report). The year/climate influenced chemical input only in sites with higher treatment levels likely due to effects on sea lice growth and reproduction. Observed differences between years are warmer surface temperature in the fall, a higher freshwater input in spring, and stronger wind conditions for 2017 and 2018 with more medication usage for these two years. The lack of significant effect of site distances calculated in zones of influence based on 24 h potential connectivity patterns highlight the need to refine the resolution of hydrodynamic processes.

## Introduction

In a recent twenty-year review of global aquaculture, Naylor et al. noted a lack of comprehensive data on the nature and extent of therapeutic use in most aquaculture sectors^1^. Patterns of chemical use in salmon aquaculture have been investigated in the United States^2^, in Norway^3–6^ , Scotland^7^, as well as in a limited manner in Canada^8,10^. Treatments and sea lice infestations can help to better understand efficiencies of management measures^4,11^ and sea lice resistance mechanisms^5,6^ also assessed through direct toxicity bioassays^5^. The high demand for effective methods of sea lice control results in rapid deployment of new strategies (including chemicals) often before extensive research into their long-term effects is completed^12,13^. Reviews and analyses of data on therapeutic approaches and technologies are important to anticipate short and long-term effects^6,9^ on resistance mechanisms and fish welfare including increased mortality^14^, as well as potential effects on non-target organisms. This highlights the importance of adequate data collection to document treatment approaches including potential link with changing environmental conditions. High sea surface temperature and salinity (> 12 °C and > 12‰) can increase sea lice growth and survival rates^15^ with more negative health effects on wild and farmed Atlantic salmon^16^.

The Canadian Aquaculture Activities Regulations require that each licensed marine finfish farm reports on usage of antibiotics and pest control products^17^. Pest control products include drugs (applied in-feed) as well as pesticides (applied using bath treatment). The information related to the chemical use in aquaculture is collected by the Canadian Aquaculture Integrated Information System (AQUIIS) with a first full year of data collection in 2016^18^. In the absence of accessible sea lice counts, chemical usage data can be used to indirectly inform on infestations with some caveats related to therapeutic approaches and potential resistance patterns. In Canada, public reporting of sea lice abundance counts in farmed salmon is implemented in the province of British Columbia^19^ while no public information on counts in Eastern Canada has been accessible until recently. As of May 2021, in the eastern province of Newfoundland and Labrador (NL), sea lice abundance numbers have started to be reported as monthly average values of sea lice per fish across all the sites belonging to one company^21^ but do not provide site specific data. A snapshot of chemical use in Canadian aquaculture sites was completed by Chang et al.^8^ by reporting data from 2016 to 2018 without considering production or environmental conditions.

The report presented here is focused on medication usage in a Canadian province (NL) and in particular on the following approved anti-sea lice medications: the bath pesticides Salmosan^®^ (azamethiphos) and Interox Paramove 50^®^ (hydrogen peroxide), and the in-feed drugs: SLICE^®^ (emamectin benzoate), IVOMEC^®^ (ivermectin), and Calicide^®^ (teflubenzuron). Here we combined publicly available data on chemical usage with information on production, stocking, site locations and environmental conditions to explore patterns of treatments, and influential environmental parameters (temperature, river input and wind). Using decision tree analyses (DTA), the objectives of this data exploration are the following: (1) the description of chemical sea lice treatments and amounts used at NL sites; (2) the effects of site characteristics (location, production) and environmental features on chemical usage.

## Results

### Number of sites and types of treatments

**Figure 1:**
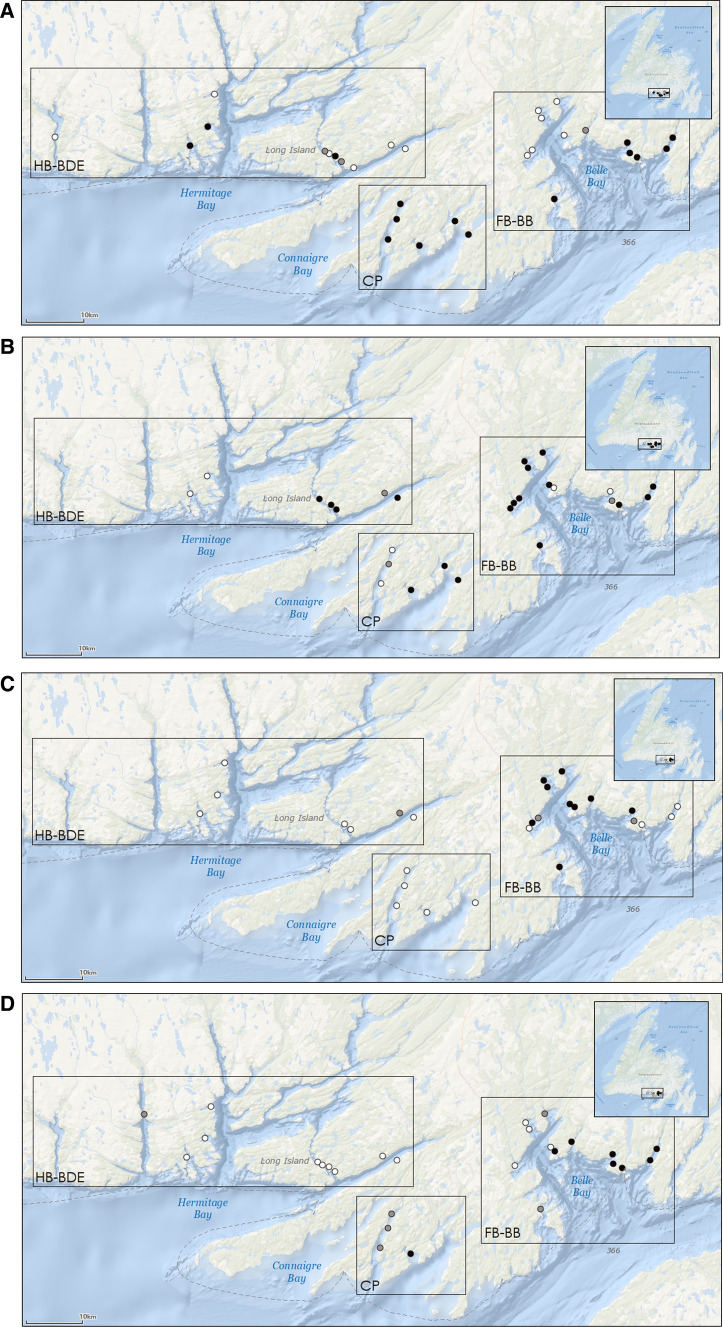
Location of all active sites on the South Coast of NL in 2016 (**A**), 2017 (**B**), 2018 (**C**), and 2019 (**D**). White dots indicate active sites with no treatments. Light grey dots indicate locations of lower anti-sea lice chemicals usage and black dots of higher usage as per DTA analyses of site usage for 4 years. Black boxes indicate bay areas as defined by 1 day water connectivity patterns^20^. FB-BB: Fortune Bay—Belle-Bay; HB-BDE: Hermitage Bay—Bay d’Espoir; CP: Connaigre Peninsula.

The aquaculture sites considered in this dataset have a total of 1139 data points of anti-sea lice chemical use. Unfortunately, the definition of “treatment” was found to be ambiguous as it may be a bath treatment of a single net-pen, a series of bath treatments on multiple net-pens, one feeding or a sequence of feeding events using medicated feed^8^. Because of these inconsistencies yearly sums were calculated. Therefore the term treatment as used in this report is in reference to the total usage of an active ingredient or a sequential usage of multiple ingredients for one site for a full year. This adds up to 65 treatments when summed per year from 2016 to 2019 for a total number of 29 aquaculture sites. These yearly treatments can be one chemical or a sequence of chemicals (Table 1). A site being in activity for 2 to 3 years, treatments can therefore be related to a single site more than once during its production cycle. Sites are located on the South Coast of NL (Fig. 1); boundaries of black boxes are based on 1 day water circulation connectivity patterns as per a previous study of the area^22^. The number of active sites per year are: 30 for 2016, and 27 for each of the remaining 3 years. The total number of active sites where anti sea lice chemicals were used are: 18 (60.0% of the active sites), 21 (77.8%), 12 (44.4%), and 14 (51.8%), for 2016, 2017, 2018, and 2019 respectively. Anti-sea lice chemicals were administered during 1 to 6 months durations with mostly summer to fall treatments (June to October with seldom treatments in November and December).

**Table 1:** Counts of treatments per year at sites (*n* = 29) where anti-sea lice medications and/or successions of chemotherapeutants were used from 2016 to 2019 in NL.

Treatments	Total counts	Counts in 2016	Counts in 2017	Counts in 2018	Counts in 2019
Azamethiphos only	9	1	2	1	5
EMB only	5	2	0	2	1
Ivermectin only	4	4	0	0	0
hydrogen peroxide only	0	0	0	0	0
EMB + Azamethiphos	27	9	8	5	5
Ivermectin + Azamethiphos	4	0	4	0	0
Ivermectin + EMB	1	0	1	0	0
EMB + Azamethiphos + hydrogen peroxide	8	0	2	3	3
Ivermectin + Azamethiphos + hydrogen peroxide	2	0	2	0	0
Ivermectin + EMB + Azamethiphos	4	2	2	0	0
Ivermectin + Azamethiphos + EMB + hydrogen peroxide	1	0	0	1	0
Total number of treatments with Azamethiphos	55	12	20	10	13
Total number of treatments with EMB	46	13	13	11	9
Total number of treatments with Ivermectin	16	6	9	1	0
Total number of treatments with hydrogen peroxide	11	0	4	4	3
Total number of treatments per year and for the 4 years	65	18	21	12	14

Table 1 reveals that most compounds other than hydrogen peroxide were used individually to treat sea lice infestations per year but that multi-chemical treatments are preferred. The most commonly used combination of products is EMB and azamethiphos. Site owners have been using EMB and azamethiphos during the full 4 years with a decline in usage of ivermectin in the past 2 years. There was no usage of teflubenzuron in NL as per the consulted dataset.

### Fish stocking data and active ingredients calculations

No statistical differences between years were observed in numbers of fish per site with treatments (Table 2) after completing Kruskal–Wallis ANOVA on ranks (*P* = 0.727). Similarly, no differences in size at stocking were noted between years (One way ANOVA, *P* = 0.268); the average size at stocking during the 2016–2019 at sites was: 168.0 ± 47.2 g. Median values as well as min and max of estimated treated biomass per site, as calculated using the thermal unit growth, are also noted in Table 2.

**Table 2:** Numbers of fish stocked at sites with sea lice treatments as well as at all active sites including estimated treated biomass at sites per year from 2016 to 2019 in NL (medians and min and max values).

Fish numbers	2016	2017	2018	2019
Median numbers of fish stocked per site with treatments	450,873 306,758–820,877 (*n* = 18)	473,886 329,320–918,265 (*n* = 21)	439,235 132,868–599,579 (*n* = 12)	432,711 132,868–896,551 (*n* = 14)
Medians numbers of fish stocked per site at all active sites (with or without treatments)	478,384 94,324–820,877 (*n* = 30)	445,184 306,758–918,265 (*n* = 27)	467,424 132,868–918,265 (*n* = 27)	486,190 132,868–896,551 (*n* = 27)
Medians of estimated standing biomass at treatments (kg) per site	225,711 94,104–477,453 (*n* = 18)	202,070 37,589–324,703 (*n* = 21)	214,760 20,656–395,722 (*n* = 12)	212,917 46,504–723,076 (*n* = 14)

The number n between brackets indicate the number of sites.

**Figure 2:**
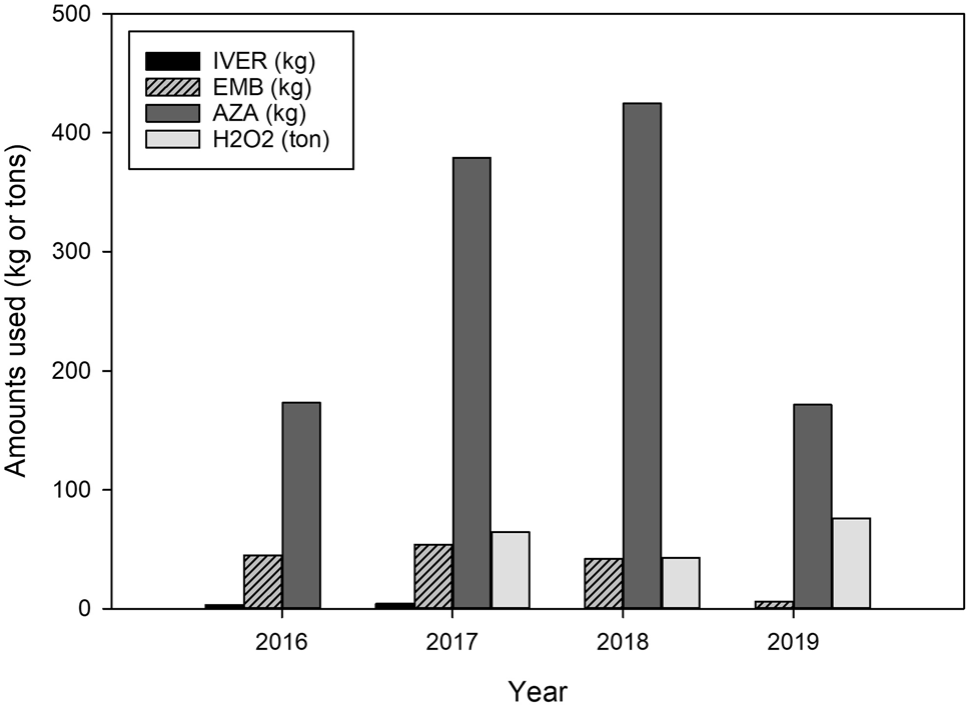
Total amounts of anti-sea lice chemicals used per year. Amounts for Ivermectin (IVER), EMB, Azamethiphos (AZA) are expressed in kgs. Hydrogen peroxide (H2O2) amounts are in tons.

The weights of active ingredient (AI) per estimated biomass were calculated for every date (used for evaluating growth from stocking) of chemical usage. For every year these rates were summed for each site. Medians of these numbers were calculated as well as minimal and maximal amounts (Table 3). Values indicate a wide range of usage per biomass for all the anti-sea lice products considered. Evidently, values depend on the therapeutic/recommended dose required for every chemical and are not directly comparable especially when considering in-feed treatments versus bath exposures. In term of amounts deposited in the environment, hydrogen peroxide had the highest weight of AI followed by azamethiphos, EMB and ivermectin. There is an overall trend of reduced chemical usage in the last year for all compounds except hydrogen peroxide (Fig. 2).

**Table 3:** Median usage of individual chemicals i.e. active ingredients per site (medians and min–max between brackets) from 2016 to 2019 expressed as total amounts and per fish weight (kg).

	Ivermectin (*n* = 16)	EMB (*n* = 46)	Azamethiphos (*n* = 55)	Hydrogen peroxide (*n* = 11)
Recommended treatment regimes for every chemical for one infestation	0.05 mg/kg administered twice weekly within a 1-week interval to 0.2 mg/kg every 2 weeks^81^	50 μg/kg fish·day-1 for 7 days^26^	100 µg/L for 30–60 minutes^35,36^	1.2–1.8 g/L for 20 minutes^28,37^
Median rates of usage for one year of treatment (mg or g/kg of estimated treated biomass per site)	4.1 mg/kg (1.3–6.8)	8.5 mg/kg (1.3–51.1)	73.8 mg/kg (4.5–424.9)	115.4 g/kg (16.7–358.5)
Median amounts (kg) of active ingredient per year and per site	0.46 kg (0.02–0.79)	1.36 kg (0.08–13.73)	13.82 kg (0.75–70.06)	10,560.0 kg (3600.00–39,980.40)

The number *n* between brackets indicate the number of total treatments per year per site.

### Decision tree analyses

**Figure 3:**
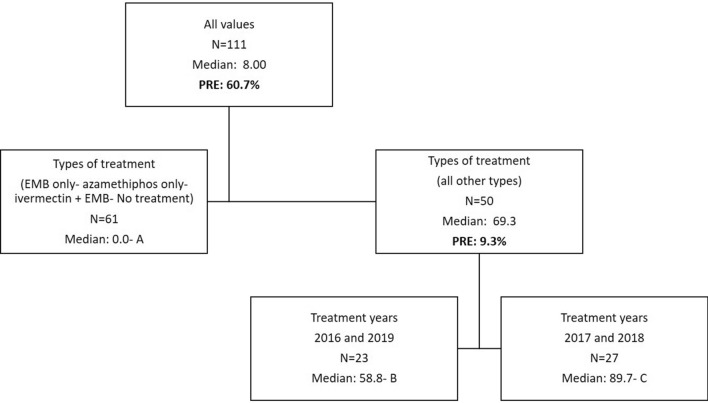
DTA with sums of normalized rates of multi-chemical usage as a dependent variable. Predictors include: Year, Bay, company, usage of lumpfish, numbers of fish, types of treatment, stocking year, and median distance to other sites. The total PRE of this tree is 70.0%.

The dependant variable for the DTAs is the rate of AI per biomass after normalization for individual chemicals when used alone during a year. When more than one chemical was used to treat sea lice for a given year these normalized rates were summed. This sum is only a proxy/index for total anti-sea lice product usage so the numbers found in the DTA figures should be considered as such. DTAs were completed with data encompassing all sites in activity whether treated or not (usage would be equal to zero for non treated sites) for a total dataset of *N* = 111 yearly entries. The following predictors were tested: Year (categorical; 4 years), Bay (categorical; 3 bays), company (categorical; 4 companies), usage of lumpfish (categorical, Yes/No), numbers of fish (continuous), types of treatment (categorical; 11 combinations as per Table 1), stocking year (categorical; stocking year one, two or three) and median distance to other sites (continuous). DTAs were tested using all the predictors. The following factors did not emerge as predictors after pruning: median distances, numbers of fish, bay, usage of lumpfish as well as stocking year. The median distance between sites during all years of treatment within bays is 15 km (4–57 km range). Nodes are designed by letters: A, B, and C, in the figures (Figs. 3, 7, 8, 9). The proportion of variance explained are also added in the figures for every split and the total variability of a tree is noted in the figure legend.

Some types of treatment resulted in lower amounts of AI being used per biomass (Fig. 3). Data as per node A includes 15 treatments at 12 sites and 46 instances of no yearly anti-sea lice chemical usage at 28 sites. The treatments with less chemical amounts correspond to the usage of the following treatments: EMB only, azamethiphos only, and the combination: EMB and ivermectin. Treatment year resulted in a significant effect on chemical usage with lower amounts in 2016 and 2019 (median proxy of 58.8 vs 89.7). One way ANOVAs between nodes was significant (*P* < 0.001) with all for pairwise comparisons (A versus B, A versus C, B versus C) being significant as well (Dunn’s method; *P* ≤ 0.001 for each comparison).

**Figure 4:**
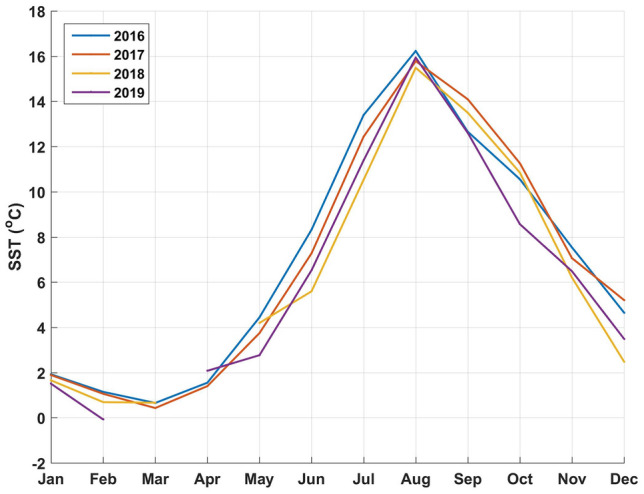
Sea Surface Temperature (SST) monthly averages for 2016–2019 of the study area (*N* = 128 in complete coverage). March 2019 and April 2018 did not return any data (no coverage).

**Figure 5:**
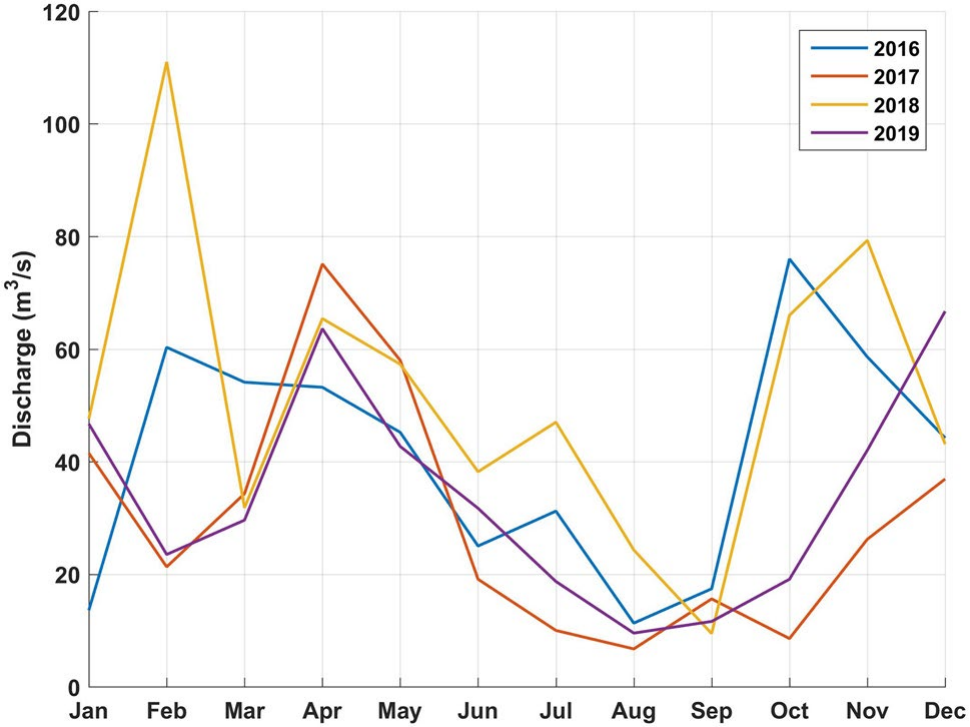
Bay du Nord river monthly averages discharge for years 2016–2019 as reported by Environment and Climate Change Canada^21^.

Monthly averages of SST, river discharge (Bay du Nord) and wind are presented in Fig. 4, 5, and 6 respectively. The data as provided are indicative of the general climatology of the full study area and are not geographically linked to a particular bay or water mass as defined in Fig. 1. The effects of environmental parameters with 12 monthly data points for both SST and river ranges and speed averages for wind data (NE, NW, SE, SW, and omnidirectional) were tested separately in the DTA. There are not enough data points to test all predictors simultaneously including all combinations without an overfitting of the data. Three DTAs were completed with either monthly values of SST (Fig. 7), river ranges (Fig. 8), or using average wind speeds from 4 prevailing directions (Fig. 9). The first predictor of influence remains the types of treatment with similar splits in the data as in Fig. 2 and evidently significantly different nodes (*P* < 0.001 following post-hoc tests as stated above). DTA indicate that the September average SST, the month of May discharge, and wind speed from the Northeast are the most influential on data.

**Figure 6:**
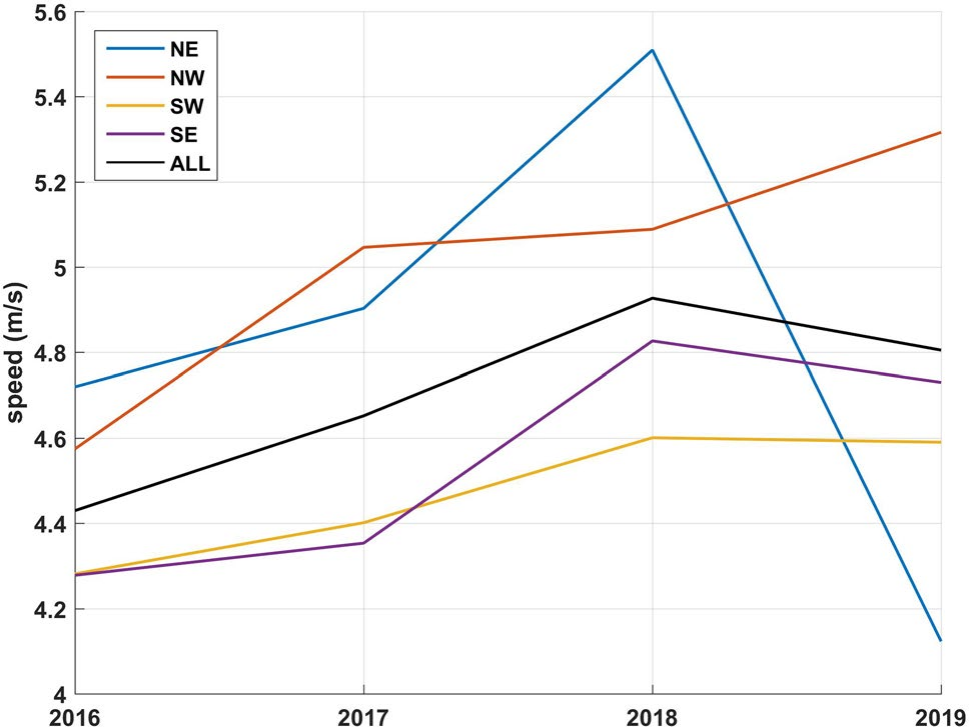
Average wind speed of the study area per main quadrant (direction from), as reported at Dog Island (Fortune Bay). NE = Northeast, NW = Northwest, SW = Southwest, SE = Southeast and ALL = omnidirectional.

**Figure 7:**
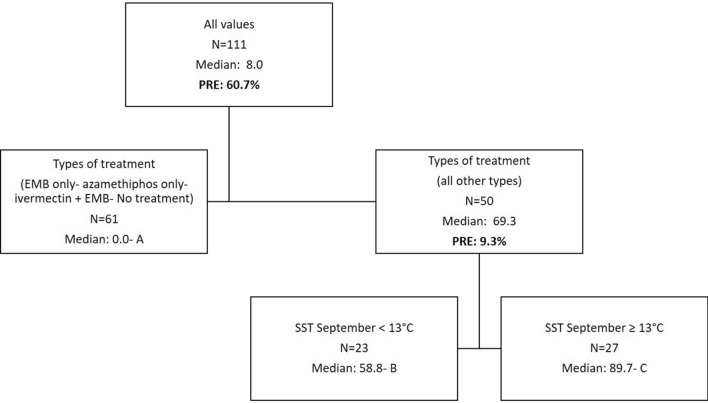
DTA with sums of normalized rates of multi-chemical usage as a dependent variable with a focus on temperature effects. Predictors include: Bay, Company, usage of lumpfish, numbers of fish, types of treatment, stocking year, SST monthly averages (January to December i.e. 12 predictors). The total PRE of this tree is 70.0%.

**Figure 8:**
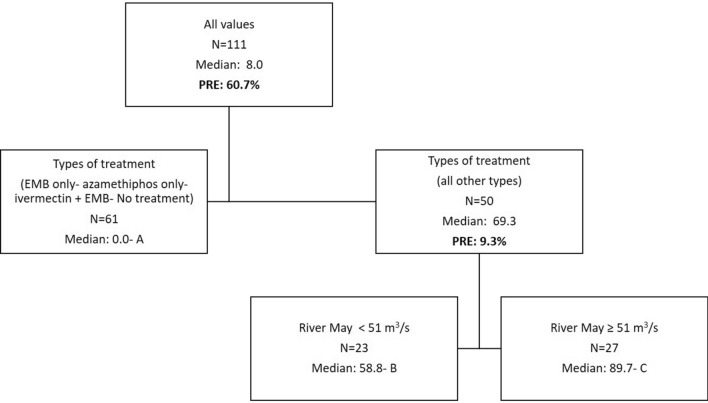
DTA with sums of normalized rates of multi-chemical usage as a dependent variable with a focus on salinity effects as per river influence. Predictors include: Bay, Company, usage of lumpfish, numbers of fish, types of treatment, stocking year, River monthly discharges (January to December i.e. 12 predictors). The total PRE of this tree is 70.0%.

**Figure 9:**
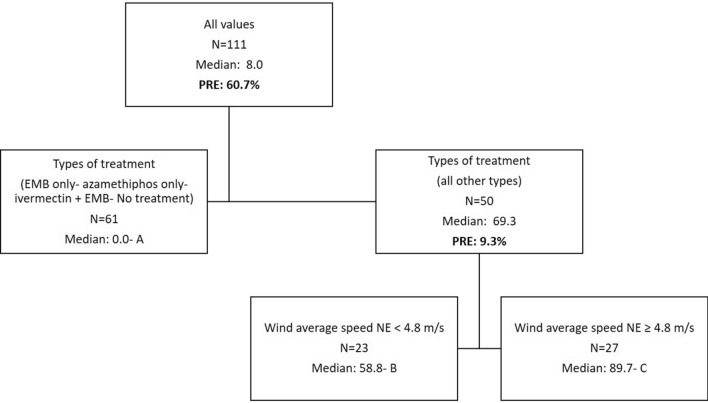
DTA with sums of normalized rates of multi-chemical usage as a dependent variable with a focus on wind effects. Predictors include: Bay, Company, usage of lumpfish, numbers of fish, types of treatment, stocking year, wind average speed (omnidirectional), wind average speed NE, wind average speed NW, wind average speed SE, wind average speed SW . The total PRE of this tree is 70.0%.

### Effect of distances between sites

**Figure 10:**
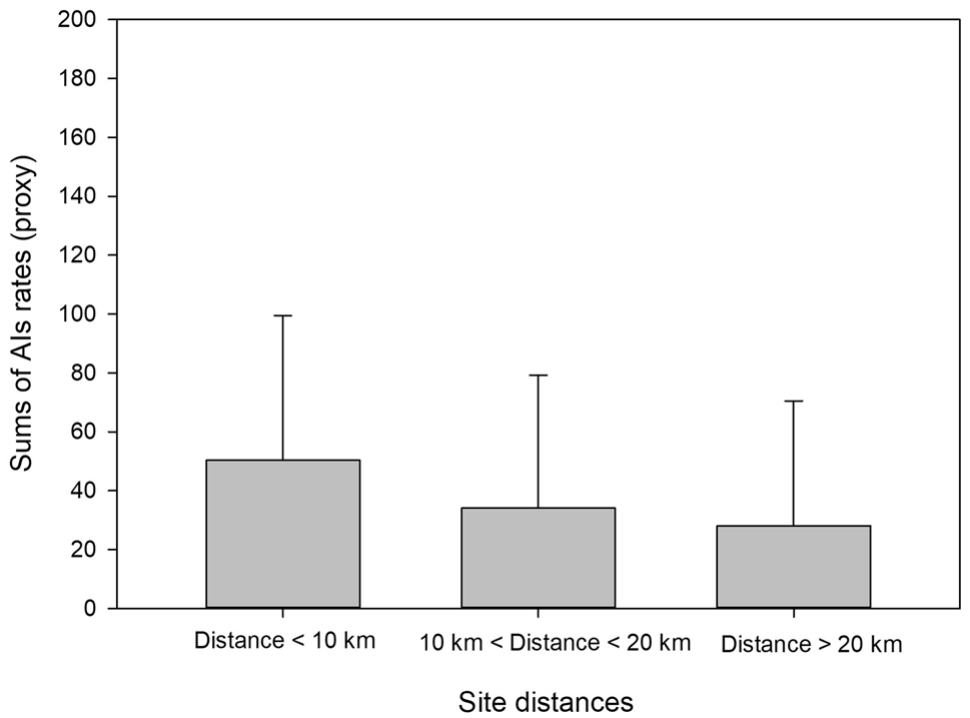
Average and standard deviations of chemical usage per site (proxy calculated as the sum of normalized active ingredient rates (AIs)) in relation to distances to adjacent treated sites. Distances are expressed as either < 10 km, between 10 and 20 km or higher than 20 km.

To further explore effect of distances we compared the sum of normalized rates for all chemicals for sites with distances subdivided in 3 ranges representative of the degree of separation of the sites. These ranges are distances < 10 km, between 10 and 20 km and > 20 km. The Kruskal–Wallis one way ANOVA on ranks (data were not normally distributed) show no statistical differences between rates for these three groups of sites (*P* = 0.195). However, we can note a tendency towards higher rates of usage for sites separated by smaller distances (Fig. 9). The median chemical proxy is 30.7 (*N* = 14 yearly data points), 10.0 (*N* = 62 yearly data points), and 1.9 (*N* = 35 yearly data points) for sites separated by less than 10 km, between 10 and 20 km and more than 20 km respectively. Figure 10 represents averages and standard deviations of chemical proxies; this was selected instead of medians for a better visualization of data.

### Effect of lumpfish treatment

As per non chemical treatments, 12 different sites used lumpfish as a biological mean to treat sea lice. Information on the usage of mechanical/thermal means of lice treatments was not available to us. The usage of lumpfish did not emerge as a predictor in the DTA. However, after testing for differences between yearly treatments of all chemical usage between sites with or without lumpfish significant disparities were found (Mann–Whitney, *P* = 0.031). Sums of normalised rates of all chemicals were lower in sites with lumpfish (*N* = 26 yearly data points; median chemical proxy = 1.0) in comparison with ones without lumpfish (*N* = 85 yearly data points; median chemical proxy = 16.6). These comparisons include instances of no treatments at all for certain years at sites with or without lumpfish.

## Discussion

### Types of treatments and rates of usage

The most commonly used chemical was azamethiphos (84.6% of treatments) followed by EMB (70.8%), ivermetin (24.6%), and hydrogen peroxide (16.9%). We can also note an overall decline in usage of ivermectin in the past 2 years. Sequential usage of chemicals is the most implemented approach though all compounds other than hydrogen peroxide were also employed as an only mean of treatment at some sites for a given year. The principle of rotating treatments has proven to be essential in order to maintain efficacies for as long as possible^3^. The most commonly used combination of products was found to be EMB and azamethiphos with 41.5% of the total number of treatments from 2016 to 2019. To prevent re-infection during the high risk period, when fish are clearing EMB, farmers may apply a chemical bath treatment as soon as juvenile sea lice are detected^10^. The frequent usage of EMB followed by azamethiphos baths suggests that this is a strategy seemingly effective in NL sites.

The standard EMB treatment regime is 50 μg/kg fish·day-1 for seven consecutive days^26^ resulting in 0.35 mg/kg for one EMB treatment. EMB usage per biomass calculated in Table 3 has values of 3 and 146 times higher than one EMB recommended treatment when contrasting min and max rates respectively. These amounts are likely related to more than one sea lice parasitic event with fish treated for recurring infestations within one year but they could also be associated with potential resistance patterns. In a review of drugs and pesticides use by the Canadian marine finfish industry in 2016–2018 Chang et al.^8^ noted that the province of British Columbia (BC) had the largest number of farms treated with EMB, while NL used the largest quantity of AI in all three years confirming to some degree our observations on high rates per biomass. These observations cannot replace actual testing of sea lice populations such as in other studies that have elegantly combined both empirical observations and toxicity data on lice^5^ but they can provide a sign. The effective concentrations of chemotherapeutant needed to remove 50% of lice for resistant strains can be more than 3–100 times higher than for sensitive strains depending on chemicals^6^. Salmon producing areas have documented the emergence of EMB resistant sea lice within 5–10 years of the product start of usage^5,6,27–29^. Within the Canadian context this has been documented in some areas such as in the Bay of Fundy^30^ with potential drivers for drug resistance in BC^10^. The North-Atlantic population of salmon lice is genetically very homogeneous^31^, as larvae can disperse over large distances prior to infecting new fish^32^. Homogeneity has also been demonstrated for resistance towards the organophosphate azamethiphos with the associated mutation widely dispersed in the North Atlantic population of lice^33^. However, another study on UK, Norway and Irish sea lice samples suggest that it is possible to discriminate between nearby *L. salmonis* populations given suitable marker selection approaches, and that such differences have an adaptive basis^34^. Further work is needed to investigate resistance in the regional context of our study and in Canada in general.

For bath pesticides, Salmosan® is used at a dose of 100 µg/L for 30–60 min in well boats and tarps^35,36^ while hydrogen peroxide (AI of Paramove^®^) has a registered dosage of 1.2–1.8 g/L for 20 minutes^28,37^. The values in Table 3 are expressed per biomass and without having the appropriate details on volume, administration routes (tarp versus well boat) it is difficult to comment on intensity. In addition, we have not found values expressed per weight in the literature to adequately contrast usage.

In term of deposited amounts in the environment hydrogen peroxide had the highest weight of AI (Fig. 2). Unlike the other compounds considered in this report, hydrogen peroxide should not at all accumulate in the environment as it splits quickly into water and oxygen, and is therefore considered environmentally benign^37^. However, recent findings document potential short-term toxicity on non-target species present around farms^38–40^. We can note that overall chemical input in the environment was reduced in 2019 (not for hydrogen peroxide) and that this trend has not been consistent within the 4 years. In Norway, Overton et al.^3^ documented that bathing with pesticides and hydrogen peroxide were reduced from 2015 to 2017, with the introduction of thermal and mechanical approaches to fill the void. In the data available to us, we have no detailed information on mechanical and thermal means though their usage has been described in industry press releases while another non-medicinal alternative, i.e. cleaner fish, considered in this study resulted in lower usage of chemicals. A recent analysis of more than 500 farms in Norway suggests that louse removal efficacy of cleaner fish was patchy in space and time^41^. The sustainability of cleaner fish usage due to issues in fisheries quotas and welfare have also been of concern^29,42^ and remain to be better studied in the Canadian context. Additionally, a decrease in the annual production of cleanerfish has been reported in recent years in Scotland^43^.

### Effect of treatments, year, and environmental variables

Site production numbers do not appear as influential predictors in the DTA. This could be influenced by the fact that overall biomass at treated sites were not statistically different between years despite the existence of a range of production values. These values are not reflective of an overall production as there was a slight decline in production tonnage after 2016 ^44–46^.

The first split in the DTA is determined by some types of treatments but mostly whether sites were treated or not and explains 60% of the variance in the total usage of anti-sea lice chemicals. When examining the sites with lower treatments (*n* = 12 sites) and no treatment at all, we can note that these are not the same locations during the different years (Fig. 1A–D), that they have similar median distances to other sites as well as comparable numbers of fish as for the other 50 treatments. Among these yearly data entries 31.1% of sites had lumpfish for the lower and no treatment group versus only 14.0% in the higher treatment group. This could have influenced the data splitting in the first node but this effect does not fully explain the resulting node as the lumpfish presence/absence is not a predictor in the DTA. Larsen and Vormedal^4^ noted that some production sites were able to keep lice levels low with fewer treatments, whereas other sites had high numbers of lice despite large numbers of treatments. In our study, only 2 sites have had consistently either no treatments or lower treatments during the 4 years. This highlights the potential effect of other farm-related and/or environmental parameter likely requiring a higher data resolution than what is used in this exploratory exercise.

The year and climate were tested separately and determined similar DTA splits in the data (9.3% of the variability) with effects only on the higher treatment sites (Fig. 7, 8 and 9). When testing temperature effects on sea lice infestations in wild sea trouts, Vollset et al.^24^ concluded that “year” had confounding effects on statistical analyses as it likely reflects differences in climate between years as also observed by Shephard et al.^25^. The year/environmental conditions did not affect the lower and no treatment datapoints with no particular pattern in percentages of treated sites per year. This suggests that the climate effects might have enhanced existing sea lice populations but did not determine the actual presence/absence of sea lice. The higher treatment years 2017 and 2018 were characterized by warmer fall surface temperatures as noted also by DFO (2018)^47^, a higher freshwater input in spring^47,48^, and stronger NE wind conditions. Unlike some salinity thresholds^49,50^ temperature does not have a limiting effect on louse survival in the ranges observed naturally^16^. Temperature mediated growth of sea lice^51^ on the other hand likely affected the severity and recurrence of infestations and consequently the amounts of chemical input. Sea lice have been shown to mostly stay within the surface 10 meters^52^ especially during the day so the effect of SST ranges are likely significant. Temperature is a determinant of louse development rate and reproduction with seasonal patterns^12^. Godwin et al.^53^ also found that the risk of sea-louse outbreaks increases in overall high-sea-surface temperature years pointing to the importance of including climate change patterns. In our study area, the ‘spring-freshet’ discharge due to the melting of snow and ice peaks in April–May and results in water stratification^54^. Sea water freshening (salinity levels < 27–29‰), has generally a negative effect on sea lice^49,50^. Except in HB-BDE, near-surface salinity rarely reaches less than 30‰. In HB-BDE salinities lesser than 30‰ are generally found only at the surface above 5m^50^. Thus, sea-lice can still have access to favorable salinity conditions below 5 m even in years of relatively large river discharge^55^. Stratification plays a role in sea-lice behavior enhancing their drive and ability to find a host below the halocline as showed by a number of studies^56–58^. Crosbie et al.^58^ recently reported interesting differences in salinity tolerances between lice stages with strong aggregative behaviour towards the halocline layer^58^. These differences would allow the two stages (nauplii and copepodids) to survive despite limiting conditions at surface (0–5 m) such as in HB-BDE with nauplii potentially occupying depths below 5 m until they reach the copepodid stage. A modelling study^59^ integrating the findings of Crosbie et al.^58^ show that avoidance of water with low salinity need to be parameterized using probability functions while ensuring not to use too strict salinity avoidance scenarios for the nauplii stages. In term of winds, given the main orientation of the bays within a SW-NE axis, a stronger effect of the wind can be expected on the circulation when winds are blowing from those directions^22,60^. Hence, stronger winds from the NE could have resulted in larger water movement contributing to more sea lice transmission amongst sites in 2017 and 2018.

There is no significant effect of median distances to adjacent sites. However, we can note a tendency for higher chemical usage in sites that are closer together (Fig. 10). Statistical models of lice epidemiology show that the risk of sea lice infestation increases with the number of farms in an area^61–63^. Median distances as evaluated in this study including the total number of fish on a farm might not have adequately captured the site connectivity for effects to be reflected in the DTA results. The number of fish on individual farms may be less important in determining sea lice numbers than the total number of fish on all farms in an area^64^. Distance alone might not equate transmission between farms as the magnitude and direction of connections influence outcomes with regions classified as net sea lice exporters while others as net receivers of particles^62,65,66^. NL observations have shown that small scale variability is present in the bays^22^ potentially having an impact on local lice transmission requiring the need to refine the understanding of connectivity and hydrodynamic processes. In addition, the length of the computed time window even if reflective of the potential for water movement between locations might not be relevant^74^ to capture adequately infection potentials of sites as *Lepeophtheirus salmonis* need a few days to grow from the Nauplius stage to its infectious copepodid stage with temperature inversely affecting the length of time^67^. The planktonic larvae of L. salmonis are non-feeding, and survive on the finite energy reserves of their yolk sac. As the copepodid ages, its energy reserves become depleted^68^. A free-swimming copepodid may survive for many days but its ability to infect a host will likely be reduced over time^74^. The energy reserves declined sharply in copepodids 5 and 7 days postmolt when compared to those aged 1 and 2 days^68^. The one day water mass boundaries are likely inadequate to accurately capture lice dispersion potential.

### Data uncertainties

This analysis is based on 4 years of data; despite being informative this time-series does not allow to document long-term trends. Similarly to others^2–6,8,16,53^ it is important to stress the importance of having access to comprehensive long-term datasets to better inform on sea lice and chemical usage. In order to learn from past mistakes, the understanding of patterns preceding for example resistance development in salmon lice is crucial^5^. Some countries such as Norway having an unprecedented transparency in its aquaculture industry with regards to operational details^5^. It is important however to emphasize the need for transparency in the global industry and the reporting of trends in chemical usage wherever it is feasible. Data should include precise information on all treatments including mechanical approaches, mortality, site environmental conditions, standing biomass and more importantly site lice counts.

We have selected rates of active ingredients per biomass and not the number of treatments as a dependent variable in the exploratory DTAs considering the the lack of precision in defining a treatment. The AAR report frequency of treatments as the number of treatment periods, specified in prescriptions, over which drugs and pesticides are to be used on a farm. For example, a single 7 day prescription represents a frequency of one (DFO 2016)^69^. A 7 day prescription could be related to an entire site or a cage with no information on the number of fish treated for every prescription on the open data. Similarly to other authors^3,5^ we have chosen to focus analyses on the expression of data per biomass despite uncertainties as this remains the best approach to understand trends in drug usage. Standing biomass at treatments were calculated using the thermal unit growth initially considered for the evaluation of salmonid growth in hatcheries and used extensively on such species^70–72^. Caution is required when the approach is applied to different temperature ranges especially with temperatures far from optimal growing conditions^71^. Industry specialists manipulate growth patterns to adapt to the final type of product they are marketing^73^. Without precise data on feeding rates, the growth regimes as used in this study might be underestimated while the absence of exact mortality times may have led to an overestimation of fish numbers. Therefore we have only discussed the ranges (min–max) of AI amounts per kg.

Considering that multi-chemical approaches are dominant, summing multi-compound usage on a site and by biomass allowed a better characterization of chemical input. Sums are used as proxies that can only be partially linked to a number of lice as they are also associated with therapeutic efficiencies and resistance. In the absence of access to site specific datasets and similarly to other authors^14,51,63^ we have used regional SST and proxies for salinity measurements and water currents. These environmental parameters are indicative of the relevant features that could influence sea lice but cannot replace actual salinity and current measurements at sites^74^ and have a very coarse resolution.

## Conclusion

*Objective 1* Sequential chemical treatments are predominantly used with EMB and azamethiphos being the most prevalent with a decrease in ivermectin usage. Rates of AI per biomass for EMB might point to potential resistance patterns in the region with more information needed on sea lice testing. The usage of lumpfish (possibly in combination with other non-chemical treatments) reduces the chemical input of anti-sea lice medications and is a promising venue for treatment with careful considerations on efficiency, welfare and environmental effects found in other salmon producing areas.

*Objective 2* An effect of the year/climate influenced chemical input in sites with ongoing treatments. Climate differences are likely driven by warmer surface temperature in the fall, stronger stratification due to a higher ‘spring-freshet’, and more wind driven water dispersion resulting in more anti-sea lice medication usage in 2017 and 2018. This climate effect likely affected parasitic growth rates and infestations but did not seem to determine presence/absence of lice. In addition, we cannot exclude the effect of year differences in usage of other non-chemical strategies not documented in this report. Some sites had lower or no chemical usage pointing to lower sea lice infestations influenced by a combination of lumpfish presence (and maybe other non-chemical methods), very localised geographic/environmental conditions (coves) not adequately captured by the too high spatial resolution environmental data used in this study.

## Material and methods

### Data compilation

Data on anti-sea lice chemical usage were gathered using the National Aquaculture Public Reporting Data (NAPRD) open data portal (DFO 2020b), the AQUIIS database, and the NL provincial government data on production, stocking and mortality. Data collection covers the years 2016 to 2019 and was selected only in relation to open net-pen operations. The NAPRD reports^18^ do not provide details on the treatment method, or which net-pens were treated. A treatment has a date of administration and a total amount of AI for each product. Dates of usage/administration were used only for the evaluation of standing biomass at treatment in order to calculate rates of usage per kg as further detailed below.

Site owners (company), bay areas reflecting the water connectivity in the region^22^, production years (one, two or three), and production (stocking numbers and calculated biomass) were added as relevant information in the final dataset. The bay areas defined from water circulation and connectivity patterns^22^ are Fortune Bay—Belle-Bay (FB-BB), Hermitage Bay—Bay d’Espoir (HB-BDE) and Connaigre Peninsula (CP) (Fig. 1). The bay areas are based on ocean currents measured at various locations for periods ranging from 26 to 235 days. These currents were used to compute the displacement of particles released from different locations for numerous 24-h windows^22^. Types of treatments (one chemical or a sequential use of chemicals) and whether cleaner fish were used or not during the year were also added as categorical data to the database. Data on lumpfish presence on sites were obtained from introduction and transfers requests submitted by companies to the Department of Fisheries and Oceans (DFO).

Only two main companies are presently operating; between 2016 and 2019 some sites had a transfer of ownership so we have included all initial owners to potentially capture different husbandry approaches. To account for density of sites per bay per year a median distance was calculated for every site by taking into account all the distances to adjacent sites in production and with treatments within a given oceanographic region (BB-FB, CP, and HB-BDE; black boxes in Fig. 1).

### Environmental parameters

Sea surface temperatures (SST) data were obtained from the National Oceanic and Atmospheric Administration (NOAA) Pathfinder 5.3 reanalysis; a global, 4 km horizontal resolution, daily dataset^75^. The data were first extracted for the region of interest and cleaned up using a high resolution coastline^54^ and a buffer of 2 km (half a pixel size) to remove potential land contamination. Data were then spatially averaged (128 pixels/data points available) to obtain daily series averaged monthly for each treatment year.

River discharge data were obtained from the Water Office of Environment and Climate Change Canada^23^. Monthly averaged data from two rivers were available for the time frame of this study: Conne river, in HB-BDE and Bay du Nord river in FB-BB. Since Conne river has a much smaller watershed than Bay du Nord river (about 100 km^2^ vs. 1200 km^2^, respectively) and shows a comparable seasonal cycle^54^ only Bay du Nord data were used and are considered representative of the overall freshwater discharge flowing into the bays. Monthly river discharge was calculated for each treatment year and used for statistical analyses. Wind data were obtained from a weather station recently deployed in Fortune Bay (Dog Island)^76^. Dog Island available data were first filtered to remove outlier data and then resampled at 10 min interval (smallest interval used in all the available time-series). Yearly and seasonal average wind speed were then calculated from these filtered time-series to be used for statistical analyses. Average wind speed was provided as omnidirectional and from four prevailing wind directions. The choice in prevailing wind directions was guided by the main general orientation of the bays which is a Southwest-Northeast axis, and were thus defined as: Northeast (NE), Northwest (NW), Southwest (SW) and Southeast (SE) quadrants.

### Standing biomass calculations

Growth calculations were completed for every site using initial stocking data (numbers and dates) and fish size at stocking (80 to 250 g) as well as treatment dates. There was uncertainties in evaluating the timing of mortalities and therefore we considered the provided initial stocking numbers to evaluate the biomass at treatment dates in most cases. Some sites with important mortalities had detailed data; these were taken into account in final calculations of standing biomass at treatments.

The thermal unit growth^70–72^ was used for growth evaluation using the following formula: W_t_ = (W_i_^0.33^ + ((T/1000) × t))^3^.

W_t_ = the weight after time t in days; Wi = initial weight; T = temperature in degrees Celsius.

Previous data on temperature monthly averages of the upper 0–20 m for each bay^54^ were used to evaluate the weight change per month for every date of chemical application. These calculations were completed for every usage of an AI and total biomass evaluations were used for calculating rates.

### Rates of active ingredients use and their sums

For every product, a rate per kg of fish was calculated by dividing the amounts of AI by the standing biomass at every usage as estimated above. These rates were added for a full year usage for every single product giving a value of usage of a compound per year per kg of fish. These values are detailed in Table 3.

For the DTAs, therapeutic doses being different for every chemical (values vary from the mg to the g per kg), the AI amount for each drug or pesticide was normalized by dividing by the maximum usage of a chemical X in all the sites with reported chemical treatments. Each value was then expressed as a percentage of a maximum. This normalization allowed the addition of all the different AIs used at a site to reflect the combination of chemicals employed sequentially by industry. This sum was used as a proxy/index for total anti-sea lice product usage (all chemicals combined) per biomass and selected as a dependent variable in the decision tree analyses.

### Statistical analyses

Prior to completing comparisons F-tests were done to ensure equality of variances. Comparisons of numbers of fish at sites with sea lice treatments between years as well as ranges of distances between sites were completed using Kruskal–Wallis ANOVA rank analyses (data were not normally distributed). Similarly, Kruskal–Wallis ANOVA rank analyses were used with pair-wise Dunn’s method testing when comparing tree nodes of chemical usage after data splitting. Comparison of size at stocking (normally distributed) was completed using ANOVA. The Mann–Whitney test for non-normal distributions was used to examine the effect of lumpfish on chemical use in sites with or without cleaner fish (SigmaPlot 13, Systat Software Inc.).

We used DTAs with sums of normalized rates per biomass of all used chemicals as a dependent variable. In the DTA, hierarchical relationships are formulated between one response (i.e. dependent) variable and several predictor (i.e. independent) variables by dividing a data set recursively into smaller, increasingly statistically homogeneous portions^77^. The final result constitutes a division of the original data set into mutually exclusive and exhaustive sub-sets defined as nodes and can include both continuous and categorical variables^77,78^. The initial list of categorical predictors included Bays, companies, types/names of chemicals used, whether lumpfish was used or not (YES/NO). Continuous predictors were numbers of fish, biomass (in kg), monthly SST and river discharge parameters as well as average wind speeds from different directions.

To better understand the potential effect of environmental conditions (temperature, salinity and wind), decision trees were completed by replacing the predictor Year with the representative measures of temperature, freshwater input or wind. Year would have a confounding effect on the final tree as environmental parameters of interests are the results of the climatic effects during a particular treatment year.

Decision trees were generated using the “rattle” package available in R^79^ based on decision tree induction algorithms founded in Quinlan^80^. The splitting criterion was expressed as proportional reduction in error (PRE). The PRE constitute the proportion of variance explained; PRE was evaluated for each node as well as for the entire tree model^77^. Stopping criteria were selected with a minimum PRE of 0.04 (4.0% variance explained) required for a split and a minimum size of *n* = 10 for splitting and *n* = 5 for final nodes to avoid reporting effect of outliers. Traditional decision tree algorithms can suffer from overfitting^79,81^ and do not include statistical significance testing to distinguish between significant and insignificant improvements in the information measure^81^. Therefore, to ensure significance, nodes were statistically compared using traditional ANOVA followed by post-hoc pairwise comparisons with Holm-Sidak Method if applicable. When data were not normally distributed a Kruskal–Wallis ANOVA followed by post-hoc pairwise comparisons with Dunn’s method were completed as stated above.

## Data Availability

Data on drugs and pesticides usage were accessible through the open data portal (DFO (Department of Fisheries and Oceans). National Aquaculture Public Reporting Data. https://open.canada.ca/data/en/dataset/288b6dc4-16dc-43cc-80a4-2a45b1f93383 (2020b)). Additional access to detailed treatment data (dates) was provided through access to the metadata underlying summaries presented on the web site. Data are available from the authors upon reasonable request and with permission of DFO aquaculture policy and management. Data on stocking and fish numbers were accessible through the DFO and DFA data internal repository; restrictions apply to the availability of these data as they would require the industry authorisations for access to site specific information. Temperature data (for growth calculations) were extracted from Donnet S. et al. (2018a) and (2018b). SST, wind and river range data were also accessible on line and/or through detailed research datasets as specified in the material and methods. These datasets are available from the corresponding author on reasonable request.
